# Stream segregation in the anesthetized auditory cortex

**DOI:** 10.1016/j.heares.2015.07.004

**Published:** 2015-10

**Authors:** Chris Scholes, Alan R. Palmer, Christian J. Sumner

**Affiliations:** a MRC Institute of Hearing Research, University Park, Nottingham, NG7 2RD, UK; b School of Psychology, University of Nottingham, Nottingham, NG7 2RD, UK

**Keywords:** Auditory cortex, Neuron, Auditory stream segregation, Adaptation, Anesthesia, BF, best frequency, SU, single unit, MU, multi-unit, VS, vector strength, PSTH, post-stimulus time histogram, FD, frequency difference, PR, presentation rate

## Abstract

Auditory stream segregation describes the way that sounds are perceptually segregated into groups or streams on the basis of perceptual attributes such as pitch or spectral content. For sequences of pure tones, segregation depends on the tones' proximity in frequency and time. In the auditory cortex (and elsewhere) responses to sequences of tones are dependent on stimulus conditions in a similar way to the perception of these stimuli. However, although highly dependent on stimulus conditions, perception is also clearly influenced by factors unrelated to the stimulus, such as attention. Exactly how ‘bottom-up’ sensory processes and non-sensory ‘top-down’ influences interact is still not clear.

Here, we recorded responses to alternating tones (ABAB …) of varying frequency difference (FD) and rate of presentation (PR) in the auditory cortex of anesthetized guinea-pigs. These data complement previous studies, in that top-down processing resulting from conscious perception should be absent or at least considerably attenuated.

Under anesthesia, the responses of cortical neurons to the tone sequences adapted rapidly, in a manner sensitive to both the FD and PR of the sequences. While the responses to tones at frequencies more distant from neuron best frequencies (BFs) decreased as the FD increased, the responses to tones near to BF increased, consistent with a release from adaptation, or forward suppression. Increases in PR resulted in reductions in responses to all tones, but the reduction was greater for tones further from BF. Although asymptotically adapted responses to tones showed behavior that was qualitatively consistent with perceptual stream segregation, responses reached asymptote within 2 s, and responses to all tones were very weak at high PRs (>12 tones per second).

A signal-detection model, driven by the cortical population response, made decisions that were dependent on both FD and PR in ways consistent with perceptual stream segregation. This included showing a range of conditions over which decisions could be made either in favor of perceptual integration or segregation, depending on the model ‘decision criterion’. However, the rate of ‘build-up’ was more rapid than seen perceptually, and at high PR responses to tones were sometimes so weak as to be undetectable by the model.

Under anesthesia, adaptation occurs rapidly, and at high PRs tones are generally poorly represented, which compromises the interpretation of the experiment. However, within these limitations, these results complement experiments in awake animals and humans. They generally support the hypothesis that ‘bottom-up’ sensory processing plays a major role in perceptual organization, and that processes underlying stream segregation are active in the absence of attention.

## Highlights

• We recorded responses of cortical neurons to sequences of tones under anesthesia.
• Fully adapted responses correlated reasonably with perceptual stream segregation.
• Responses to tone sequences were weak during rapid tone presentation (>12 Hz).
• Adaptation under anesthesia is too rapid to account for perceptual ‘build-up’.
• Neural correlates of stream segregation are not reliant on top-down influences.

## 1 Introduction

One of the most impressive outcomes of processing in the auditory system is the separation of elements in the complex acoustic waveforms at each ear and their recombination (grouping) into relevant perceptual objects (see Bregman, 1990 for a comprehensive account). This ‘auditory scene analysis’ can be split into a ‘primitive’, hard-wired stage and a ‘schema-based’ stage which involves modulation by experience, prior stimuli and attention (for reviews see Carlyon, 2004; Snyder and Alain, 2007; Winkler et al., 2009; Shamma and Micheyl, 2010). However, the interplay between primitive (bottom-up) and schema-based (top-down) processing is still a matter of debate (Macken et al., 2003; Thompson et al., 2011; Snyder et al., 2012; Spielmann et al., 2014).

Many studies of auditory scene analysis focus on stream segregation: the way that a temporal sequence of sounds is grouped or split perceptually. This has been most thoroughly investigated for pure tones, where the resulting perceptual organization depends on the proximity of the tones in frequency and time (van Noorden, 1977). These perceptual effects are reflected both in responses to tone sequences in central auditory neurons in animals (Fishman et al., 2001; Kanwal et al., 2003; Bee and Klump, 2004, 2005; Micheyl et al., 2005; Pressnitzer et al., 2008; Elhilali et al., 2009; Bee et al., 2010; Christison-Lagay et al., 2015) and non-invasive measures in humans (such as far-field electrophysiological responses to individual tones: Snyder et al., 2006).

Neurophysiological studies have revealed that tones in perceptually separate streams are, qualitatively at least, represented by different populations of neurons. Recordings from auditory cortex of monkeys (Fishman et al., 2004), bats (Kanwal et al., 2003), birds (the avian analogue Field L: Bee and Klump, 2004; Itatani and Klump, 2014), ferrets (Elhilali et al., 2009), and from the auditory brainstem of guinea pigs (Pressnitzer et al., 2008) all show that tones with small frequency differences (FDs) stimulate overlapping populations of neurons whilst tones with large FDs tend to stimulate different populations. This is at least in part due to frequency selectivity established in the cochlea (Rose and Moore, 2000). However, the degree of overlap between stimulated populations also decreases with increasing presentation rate (PR), similar to the perceptual dependence (e.g. Fishman et al., 2004).

The second important perceptual effect is the ‘build-up’ of stream segregation. A tone sequence is always perceived as a single stream initially; the perception of segregated streams only builds up over several seconds, with the rate of build-up also dependent on the FD and PR (Bregman, 1978; Anstis and Saida, 1985). Neural responses decrease over time and this ‘adaptation’ has been posited as a neurophysiological correlate of build-up (Micheyl et al., 2005). In this paper, we use adaptation to refer to the general decrease in neural response with repeated tone presentation, and we use ‘suppression’ to refer to the decrease in the response to a tone that is preceded by another (relative to when that tone is presented alone). In neither case do we imply an underlying mechanism. The neurophysiological build-up of streaming has been characterized in single units in the auditory cortex of awake macaque (Micheyl et al., 2005), in multi-units in field L of the awake starling (Bee et al., 2010) and also at lower levels of the auditory system of the anesthetized guinea pig, in single units of the ventral cochlear nucleus (Pressnitzer et al., 2008).

The build-up of stream segregation and the dependence on FD and PR are often held to result from ‘primitive’ processing (Macken et al., 2003). However, ‘schema-based’ processes such as attention (van Noorden, 1977; Bregman, 1990) and spontaneous perceptual changes (flipping between two different perceptions of the same stimulus: Pressnitzer and Hupe, 2006) HuHupoe clearly influence stream segregation. Similarly, non-invasive imaging studies demonstrate both bottom-up (Snyder et al., 2006; Sussman et al., 2007) and top-down effects (Hillyard et al., 1973; Alain and Woods, 1994; Gutschalk et al., 2005; Cusack, 2005; Snyder et al., 2006; Bidet-Caulet et al., 2007; Hill et al., 2011; Lakatos et al., 2013).

Previous neurophysiological studies of auditory streaming in the cortex were conducted in awake animals, but, with one exception (Micheyl et al., 2005) the animals were listening passively and attention was not controlled. Here, to identify those elements of streaming that are based only upon primitive (bottom-up) processing, we characterize streaming in neural responses in the auditory cortex of anesthetized animals, where there are no effects of attention. The current study is partly at the single unit level and hence complements the previous studies in cortex which reported the combined responses of several neurons at a time using either multi-unit (Fishman et al., 2001, 2004; Bee et al., 2010) or current source density analysis of local field potentials (Fishman et al., 2001, 2004). It also provides a basis for comparison with other studies of cortical adaptation and suppression in anesthetized animals (e.g. Ulanovsky et al., 2004; Scholes et al., 2011; Taaseh et al., 2011).

## 2 Methods

### 2.1 Subjects and surgical procedures

Experiments were performed on 12 pigmented guinea pigs of both sexes weighing 370–737 g (mean 565 g). All animals were anesthetized with an intra-peritoneal injection of urethane (4.5 ml/kg in a 20% solution), supplemented with intra-muscular injections of 0.2 ml Hypnorm (Fentanyl citrate 0.315 mg/ml, fluanisone 10 mg/ml) whenever a forepaw withdrawal reflex could be elicited. A pre-medication of 0.06 mg/kg Atropine Sulphate was administered subcutaneously to suppress bronchial secretions. Each animal was tracheotomised, artificially respired and core temperature was maintained at 38 °C by means of a heating blanket. The animals were placed in a stereotaxic frame with hollow plastic speculae replacing the ear bars, inside a sound-attenuating room. To equalize pressure across the tympanic membrane, the bulla on each side was vented with a polyethylene tube (22 cm long, 0.5 mm diameter). The membrane overlying the foramen magnum was opened to release the pressure of the cerebrospinal fluid. A craniotomy with a diameter of around 5 mm was performed to expose the primary auditory cortex, the dura was removed and the brain was covered with a layer of 1.5% Agar. A linear multi-electrode array, consisting of four to eight glass-coated sharp tungsten micro-electrodes was advanced together and directly into auditory cortex using a piezoelectric motor (Burleigh Inchworm IW-700/710). All experiments were conducted under license from the Home Office in the UK.

### 2.2 Acoustic stimuli and electrophysiological recording

Auditory stimuli were delivered diotically through sealed acoustic systems, consisting of modified Radio Shack 40–1377 tweeters coupled to damped probe tubes that fitted into the speculae. The maximum output level of the system was calibrated a few mm from the eardrum using a 1 mm probe tube microphone (Bruel & Kjaer 4134). This was to ensure that sound levels were consistent across experiments (±3 dB). All stimuli were generated by an array processor (TDT AP2, Alachua, FL, USA) and output at a sample rate of 100 kHz. Stimulus control was from a PC using Brainware (developed by J. Schnupp, University of Oxford). Responses from the electrodes were acquired using a Medusa Headstage and Tucker Davis RX7, sampled at 25 kHz with 16-bit resolution, and digitally filtered (300 Hz–3 kHz) and amplified (∼× 40 k). Spike waveforms and spike times were recorded to disk by Brainware. They were further analyzed off-line with Plexon (Dallas, TX) spike-sorting software to isolate action potentials from separate single units (SU) and multi-unit (MU) clusters.

### 2.3 Stimuli

We presented sequences of interleaved ABAB tones (where A and B are different frequencies), as used previously to investigate the effect of varying FD and PR (Fishman et al., 2004). Tones were 50 ms in duration with 4.5 ms linear ramps at the beginning and end. Initially, pseudorandom sequences of pure tones of varying frequency and level were presented to assess the frequency tuning properties across the electrode array. Typically, a course receptive field was followed by an iso-level function (discharge rate as a function of tone frequency) at a small range of sound levels (60–80 dB SPL), from which the conditions for the alternating tone sequences were chosen. The frequency of the A tone in the alternating sequence was chosen to be close to the best frequency (BF: frequency that elicited the largest response) of a single neuron or multi-unit cluster. The frequency of the B tone was monotonically varied in either ascending or descending frequency steps (typically 1.5, 3 or 6 semitone steps depending on the bandwidth of the neuron). The tones were presented at sound levels in the range 60–80 dB SPL, but at a fixed level for a given recording. For each condition, the stimulus was selected randomly from the range of B tone frequencies and a range of PRs (2, 4, 8, 12 or 16 Hz). Due to the limited amount of time available for single unit recording, the full range of PRs was not tested for each unit. The use of multi-electrode-arrays allowed several units to be recorded simultaneously. Although care was taken to align the electrode along an iso-frequency contour so that neurons with similar tuning properties were recorded, for some of the recorded units, the A tone was not at the BF. For the purposes of population analysis, only those units for which the A tone elicited the maximal spike count were considered.

### 2.4 Data analysis

#### 2.4.1 Spike count time windows

The duration of the driven response to a tone differed across units. Some responded only at stimulus onset whilst some responded for considerably longer than the tone duration. Some units also fired spontaneously between tones, but not necessarily at the same rate as during long periods of silence or between tones at different PRs. Therefore, in order to constrain spike count estimates to the driven spikes in response to each tone, spikes were counted within windows that were determined for each unit individually by first deriving time windows at each PR. A 128-bin peristimulus time histogram (PSTH) was constructed of the responses to single tones, calculated across the period of presentation (i.e. 250 ms for 4 Hz), across all tone frequencies. Initially, the mean and standard deviation of the spike count across all the PSTH bins was calculated. The individual bins which exceeded the mean plus *one* standard deviation formed an initial ‘binary mask’ estimating in which bins a driven response occurred. A new estimate of the mean and standard deviation was then calculated from the bins *not* exceeding this criterion. A new binary mask was then formed by the bins which exceeded the updated mean plus *two* standard deviations. The bins excluded yielded yet another mean and standard deviation estimate, and so on. The process was iterated ten times with a criterion of two standard deviations. From the final binary mask we derived a single contiguous analysis window, which was defined as the time between the first and last bins between which 80% of bins were above threshold. This last stage produced a single spike count for the response to each tone, yet excluded occasional outlying bins which were above threshold by chance. For a given unit, this calculation was performed separately for each PR. However, a single window was then chosen to derive spike counts across all PRs using the earliest start and latest end of the computed windows at any PR. This window was constrained to be no longer than the fastest PR used for a given unit.

In the majority of units this method resulted spike count windows being either shorter (<40 ms in 37 units) or longer (>60 ms in 57 units) than the tone duration. Using a fixed (50 ms) analysis window did not alter any conclusions, but it resulted in less differentiation between the responses to the tones in the different conditions, and to the no-tone reference condition, and was obviously inappropriate in many instances.

#### 2.4.2 Tone PSTHs and statistics

**Fig. 1:**
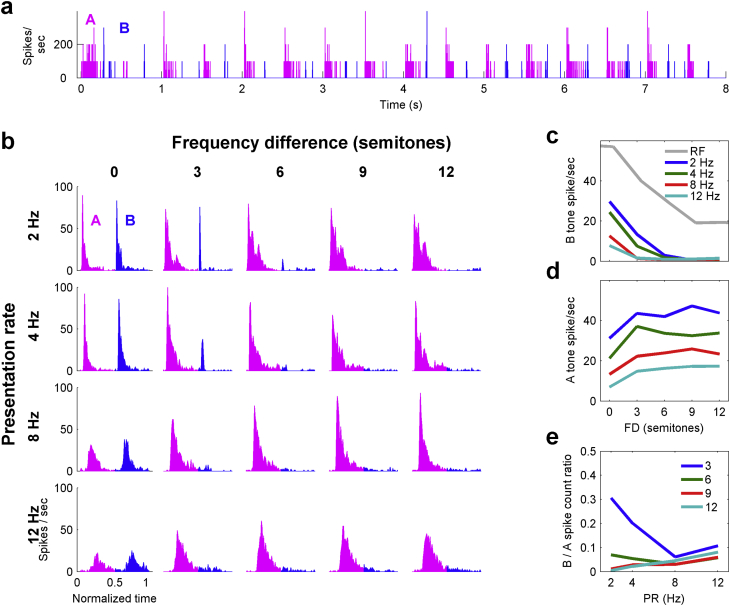
The steady-state responses of a single neuron in the auditory cortex of anesthetized guinea pig. a) PSTH of the response to a two-tone sequence with a PR of 4 Hz and FD of 3 semitones. b) Two-tone PSTHs of the steady-state (2s onwards) response to the A (2.8 kHz) and B tones as a function of FD and PR. c) The response to the B tone decreased with increasing FD and PR. d) As the FD was increased the response to the A tone increased. e) The ratio of spike counts (B/A) shows that the B tone was suppressed to a larger extent than the A tone as the PR was increased.

To visualize the dependence of steady-state responses on FD and PR we constructed two-tone PSTHs using all spikes occurring after 2 s of stimulus presentation. The width of the two-tone PSTH was defined as the presentation time for one tone pair (e.g. 1 s for 2 Hz, 0.5 s for 4 Hz) and responses were binned (100 bins) across each two-tone period. For comparison across different PRs we display the two-tone PSTHs in terms of the fractions of the two-tone presentation time (see Fig. 1).

To characterize the extent to which unit responses were locked to the tone, we constructed PSTHs with a duration of one tone (100 bins irrespective of PR). These we treated as period histograms and calculated the vector strength (VS) as defined by Goldberg and Brown (1969). The VS takes a value of 1 if all spikes occur at one precise phase (in a single bin) and 0 for a uniform distribution of phases across the duration of the tone. To assess the statistical significance of the vector strength, we used the Rayleigh test to show that a period histogram is a sample of an oriented distribution rather than a uniform distribution, using the factor 2*n*(*VS*)^2^, where *n* is the total number of spikes. A Rayleigh value larger than 13.8 indicates that the probability that the distribution is uniform is less than 0.001 (Buunen and Rhode, 1978; Mardia and Jupp, 2000).

#### 2.4.3 Statistical tests

To characterize the behavior of the neural population, ANOVAs were run on the log transformed (to approximate normality) spike counts, independently for the responses to A and B tones, with FD, PR, unit type (SU/MU) and whether high (>1.5 kHz) or low BFs (this roughly divided the dataset in two) as factors. Unit identity was included as a random factor nested within unit type.

#### 2.4.4 Adaptation of responses

To quantify the rate of adaptation of the responses to each subsequent tone in a sequence, for each stimulus condition the mean population spike counts in response to each tone, as a function of tone onset time, were fitted (using a least-square fitting procedure) with an exponential-decay function (Kiang et al., 1965; Harris and Dallos, 1979; Smith, 1979):

$$\text{C}\left(\text{t}\right)={\text{C}}_{0}+{\text{De}}^{-\text{Rt}}$$

where C(t) is the spike count for the tone starting at time *t*; C_0_ is the asymptotic spike count; D is the difference between the initial and asymptotic (predicted) spike counts; and R is the rate of adaptation.

#### 2.4.5 Signal detection model

A signal-detection-theory (SDT) based analysis was used to model how spike counts across the population might be interpreted as evidence in favor of perceiving either 1 or 2 streams of tones. Conceptually, the analysis model assumes that the evidence for perceiving 1 or 2 streams depends on the extent to which the two sets of tones are represented by different populations (“2-streams”) or a single population (“1-stream”) of neurons. Due to the experimental design, we evaluate the population of neurons which would represent only the A-tones in the 2-stream case. The assumption is that a different but mirrored population represents the B tones. If there is always a robust response to the A tone in the population, then the analysis need only consider the response to the B tone (Micheyl et al., 2005; Pressnitzer et al., 2008). As we shall see this is not always the case in our data, which partly motivated the current extension to the model proposed by Micheyl et al. to take account of the responses to both A and B tone responses, and the background spiking.

The analysis compares the population spike count distributions to the A and B tones. For each unit and each stimulus condition, spike count distributions were calculated of the responses to the repeats of the same tone (A tone or B tone, defined by their position in the sequence). Spike count distributions from individual units were normalized by dividing by the total spike count in the distribution. The distributions from individual units were then convolved together to produce a population spike count probability distribution, for each tone in each condition (see Fig. 6 following Micheyl et al., 2005).

In addition to comparing the responses to A and B tones, it is important to know whether there is any detectable response to the tones at all. To determine this, we need to compare the response to a tone with the response during a silent interval occurring at the same place in the stimulus sequence, since the responses in the silent interval between them may to some extent be dependent on the preceding tones. Therefore we computed the spike count distribution from the silent gaps where alternate tones would have been, in tone sequences presented at half the rate under consideration (or the closest available rate) with an FD of zero. In this way we were able to directly compare spike counts with and without the presence of the B tone.

The degree to which the neural responses to a particular sequence were consistent with a 1- or 2-stream organization was determined by comparing the normalized population spike count distributions in response to the A tones, B tones and silent gaps. If the responses to A and B tones were similar, the data would imply a 1-stream organization. Alternatively, if only the responses to A tones exceeded the spike rate during the silent gaps then the data clearly imply a 2-stream organization (with the presumption that another population would still be responding to the missing tones). However, if there were driven responses to both the A and B tones but the firing rates were very different, then the interpretation is less clear.

Using SDT, the decision as to whether only A tones or A- and B-tones are represented depends on a criterion spike count. In previous studies this criterion was either a free parameter used to fit psychophysical data (Micheyl et al., 2005; Pressnitzer et al., 2008) or fixed relative to spontaneous firing rates (Bee et al., 2010). Here, instead, we consider decisions overall possible criterion values and describe the range of criteria over which either 1- or 2-stream interpretations are possible (Fig. 7). First, we determined whether the tone was detectable compared to the silent gaps by setting a criterion such that 75% of the distribution of spike counts in the silent gaps were below it (i.e. 25% or lower false alarm rate). A particular tone was then considered to be represented if 75% of the spike count distribution, during the tone, fell above the criterion value. The criterion was varied systematically up to the value where the hit rate for the tone responses fell below 75%, thereby establishing a range of criterion values for which there was evidence of the tone. Note that whilst the choice of 75% is arbitrary, changing it shifts all decision boundaries in the same direction and so does not greatly affect the results.

For a given sequence of tones, the status of each consecutive pair of A and B tones was considered jointly for every criterion value, to assess whether one tone (i.e. a 2-stream percept) or both tones (i.e. a 1-stream percept) were represented. The degree to which the neural responses suggested a two-stream organization was summarized as the proportion of criterion values over which only one of the tones was represented. An average measure of streaming when neuronal responses were adapted was also computed from average spike count distributions calculated over the period 4–8s after the start of the sequence.

## 3 Results

We report the responses of 106 units (55 SU, 51 MU) in the auditory cortex of anesthetized guinea-pigs. Best frequencies (BFs) at the level of the streaming stimulus were as high as 17 kHz, but there was an emphasis on low frequencies with 50% of BFs being below 2 kHz. We presented sequences of pure tones of alternating frequency (ABAB), where the presentation rate (PR) of the tones and the frequency difference (FD) was varied systematically within each unit. For all conditions, responses to both the A and B tones adapted to a steady firing rate within around 2 s of the stimulus onset. Therefore, we first describe the properties of adapted firing rates as previously described in awake animals by Fishman et al. (2001, 2004) and Bee and Klump (2004).

### 3.1 Steady-state responses in single cortical neurons are qualitatively similar to those in awake animals

The response of a single unit to a complete tone sequence is displayed as a PSTH in Fig. 1a. Individual neurons and multi-unit clusters tended to lock their responses to each tone in the sequence, and these responses adapted as the sequence progressed. To show the response of each neuron to repeated tone pairs after the initial adaptation (the steady-state response) we summed the responses to each pair of A and B tones after the first 2 s, to create two-tone PSTHs (Fig. 1b). The two-tone PSTHs display several of the features noted in awake preparations. First, the response to the B tone decreases as the FD is increased at all PRs. Second, as the PR is increased there is a decrease in the response to both the A and the B tones. This results in weak responses at the highest PRs and smallest FDs. However, with increasing PR the response to the B tones decreases to a larger extent than the response to the A tones. Thus, at the fastest PR (12 Hz in Fig. 1b), the neuron only responds to the A tones. The response to the A tones increases as the frequency difference increases (Fig. 1d).

Overall, the responses of this neuron are consistent with a process of differential forward suppression between the A and B tone responses. The responses to A and B tones mutually suppress each other more when they are closer in frequency. As the B tone frequency moves further from the center of the neuron's receptive field, the response to the B tones weakens. Conversely, the A tones response grows with FD even though the A tone frequency is not changing, presumably reflecting a decreased suppression from the B tones. As PR increases, the response to the B tones decreases relatively more than the response to the A tones. Of course, there is no *a priori* reason to suppose that suppression cannot build up over multiple tones. An alternative way of thinking about this is that the closer the B tone frequency is to the A tone frequency the more response the tones evoke overall and this in turn drives some adaptation process more strongly. When the FD is larger, the B tone response drops and there is in turn less adaptation of the A tone. Changes in these differential responses across stimulus conditions can be characterized as the ratio (Fig. 1e) between the B and A tone responses (Fig. 1c, d).

### 3.2 Steady-state population responses are consistent with observations from cortical neurons of awake animals

**Fig. 2:**
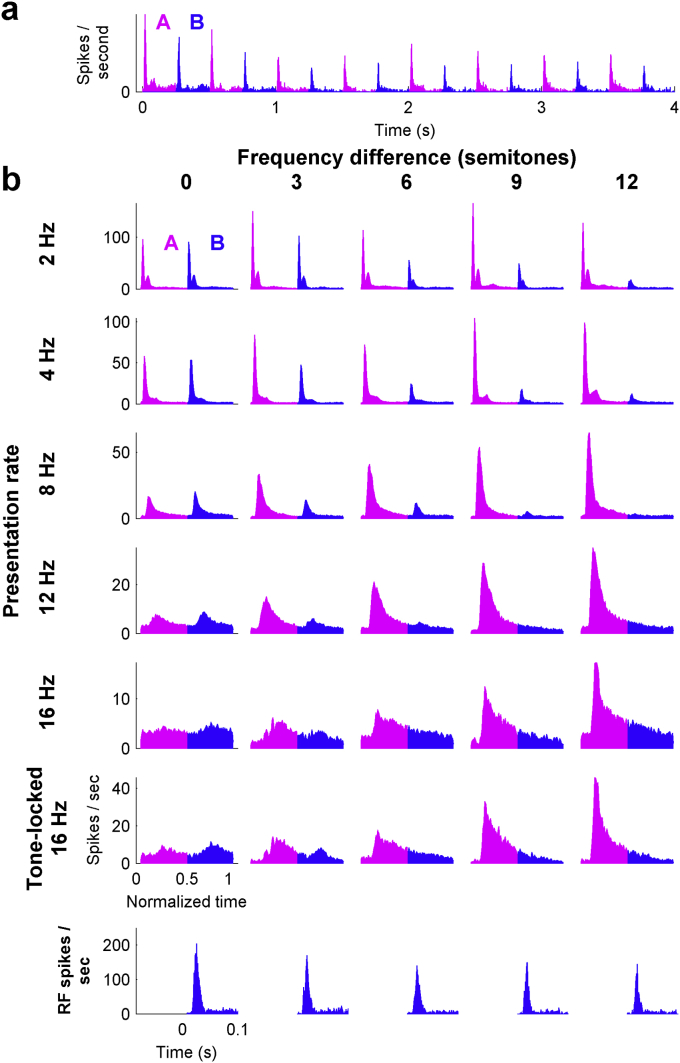
The response of the population of A1 neurons to alternating tone sequences. a) PSTH of the population responses for a tone sequence with a PR of 4 Hz and FD of 3 semitones. b) Two-tone PSTHs of the population of neurons for all conditions tested, including the result of only using those units that significantly locked at 16 Hz (second to bottom row) and the response to tones presented in isolation at the B frequencies (bottom row).

The mean population PSTH for 4s of a two-tone sequence, from all recordings (78) in which the largest response was to the A tones (39 SU, 39 MU), is displayed in Fig. 2a. The corresponding two-tone PSTHs for all conditions are shown in Fig. 2b. The trends observed in the single unit example of Fig. 1 are apparent in these average population responses. As the FD is increased, the response to the B tones decreases monotonically and the response to the A tones increases monotonically. The bottom row of Fig. 2b shows the population response to isolated B tones, presented at the same frequencies and levels as the B tones in the ABAB sequences. This shows that pure tone responses were generally broadly tuned in these cortical neurons (>1 octave). Thus, the responses to the B tones within the sequences are restricted to a narrower range of frequencies than is measured in responses to isolated tones. The lack of response to the B tones at the higher FD is not therefore a simple reflection of the response area filtering.

**Fig. 3:**
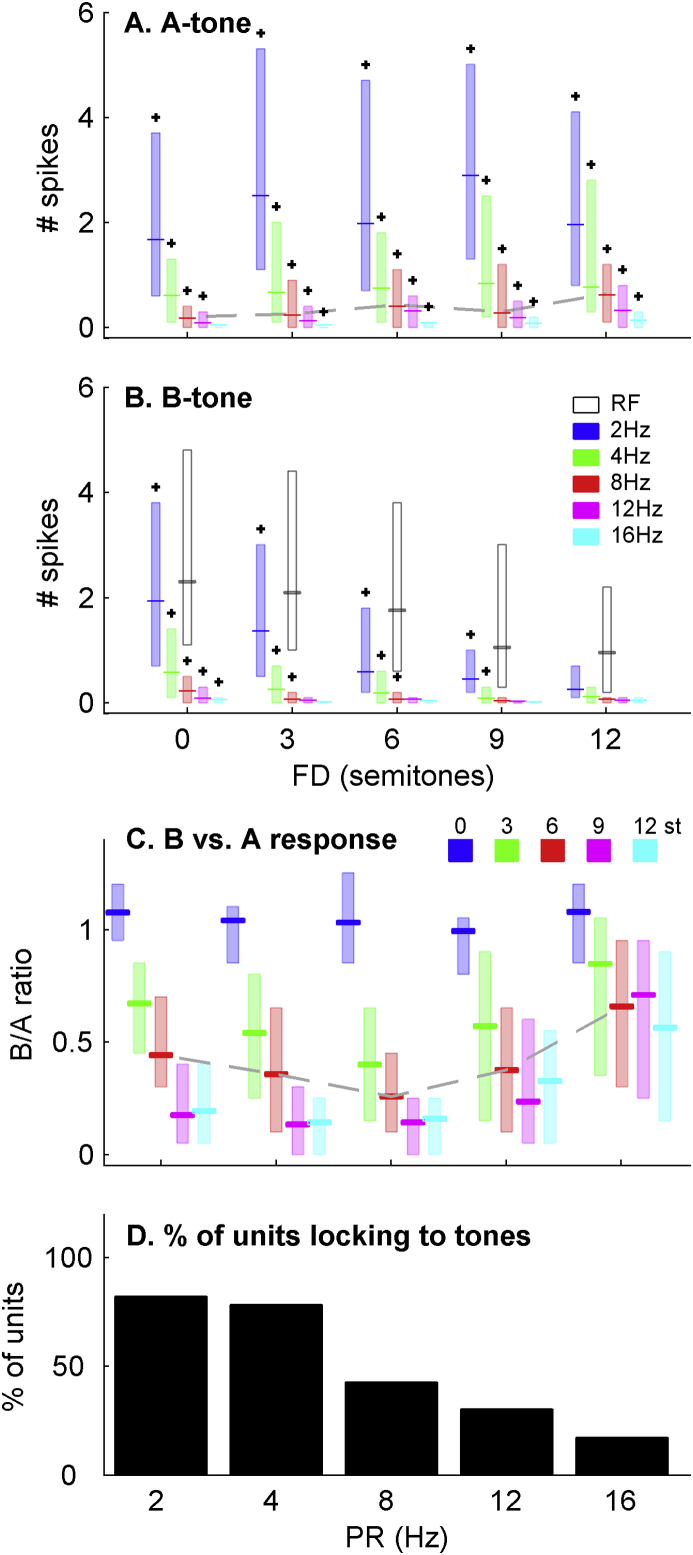
A) Median and interquartile spike counts across the population in response to each A tone. Asterisks indicate when the population is significantly driven (p < .05). The grey dashed line illustrates increase in A-tone response with FD for 8 Hz. B) Spike counts in response to the B tones. Open bars indicate the responses to isolated tones at the B tone frequencies. C) The B/A spike count ratios as a function of FD and PR. Dashed grey line illustrates the non-monotonicity of ratio as function of PR. D) The proportion of units that exhibited significant locking to the tones in the sequence decreased markedly at faster PRs.

Fig. 3A and B shows the responses to the A and B tones respectively, expressed as median and interquartile spike counts across the population of neurons. These show three main trends. First, the responses to the A tone increase as FD increases (dashed line illustrates this for 8 Hz PR). Second, responses to B tones drop with increasing FD. As in Fig. 2, this reduction is also seen in the spike counts in response to isolated tones (open bars in Fig. 3B). Third, all spike rates decrease with increasing PR. Across the population and considering all conditions, these effects of PR and FD on both A and B tone responses were all significant as was an interaction between FD and PR (all p<<0.01, ANOVA, see methods). Additionally, spike counts were higher at low BFs (p < .05). There was no effect of unit type (SU or MU). Fig. 3C shows spike counts expressed as the ratio of B tone responses to A tone responses, revealing changes in the relative responses across the stimulus conditions (significant over FD and PR, p<<0.01). A differential reduction of the B tone response relative to the A tone response (evident as a decrease in the B/A ratio with increasing PR; Fig. 3C) can be observed up to 8 Hz. At higher PRs, the A and B tone responses actually become more similar. From Fig. 3A and B, it is evident that this is because the responses to both A and B tones are weak at high PRs.

Given the low spike counts at high PRs, it is reasonable to ask whether there were in fact driven responses to tones. We compared the number of spikes elicited by the A and B tones, and the number of spikes that were observed at the periods in time when these tones were omitted (calculated from gaps in between the responses to tones at lower PRs, see methods). Driven responses to A tones were significant in all conditions, however this was not true for the B tones (Holm-Bonferroni corrected Friedman tests of each condition, p < .05; asterisks indicate significance in Fig. 3A and B). Significant driven activity for the B tones was restricted to progressively slower PRs as the FD was increased. The number of units that locked their responses to the tones also decreased as the PR increased (Fig. 3D) so at 12 and 16 Hz the response locked to the tones was only significant in a minority of units (Rayleigh criterion, p < .001). There were no differences in the proportion of SU and MUs locked to the tones (χ^2^ tests).

Thus, at a population level and in some single neurons at least, the adapted responses of cortical neurons under anesthesia are broadly similar to responses reported in awake animals. However, at high PRs responses are strongly adapted, locking to individual tones is poor and the magnitude of A tone adaptation is dependent on the B tone frequency.

### 3.3 Units not centered on the A tone frequency are generally consistent with different neural populations coding individual streams

Previous reports of neural responses to alternating tone sequences have generally focused on the case where a neuron (or group of neurons) responds maximally to the A tone. Through the use of multi-electrode arrays we recorded from several units at a time. In choosing a single fixed A tone frequency to record from several units simultaneously, there were a proportion of units (28) for which the A tone was not the most effective frequency. These units were heterogeneous in their response properties, and therefore were not analyzed as a population.

**Fig. 4:**
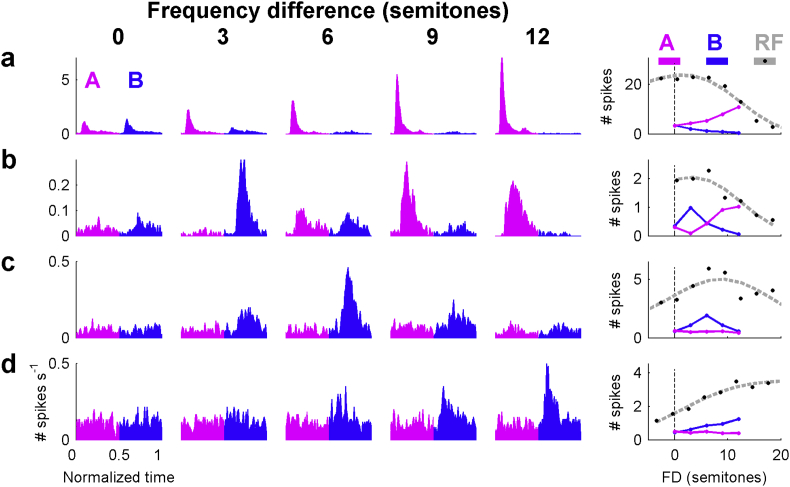
Responses to AB tone sequences are governed by the tuning of the neuron. a) Two-tone PSTHs display the characteristic pattern exhibited by a unit with the A tone (4.8 kHz, MU) set at BF. Right hand panel shows the spike count in response to A and B tones in the sequence and also to isolated tones (black). b) In this unit, the B tone dominates the response at a 3 semitone difference, however the A tone (1.6 kHz, SU) still dominates at larger semitone differences. c) This neuron responds maximally to the B tone with a 6 semitone FD; there is little response to the A tone (1 kHz, MU) at any FD, despite a robust response to isolated tones. d) In this example, the neuron CF is at frequencies beyond the range of tone frequencies used and there is no response to the A tone (0.7 kHz, MU) in the sequence.

Fig. 4 illustrates how the position of the tones within the receptive field of a neuron can influence the steady-state response of auditory cortex neurons to AB tone sequences, at a PR of 8 Hz. Fig. 4a shows another example of a unit that responds maximally to the A tone (see Fig. 1), displaying the familiar pattern of decreasing B tone responses and increasing A tone responses as the FD is increased. The right hand panel shows the spike count in response to the A and B tones across these conditions, and also shows the spike counts recorded during RF measurement. The trend seen for the B tones is consistent with the response to isolated tones in the RF, and the response to the fixed frequency A tones is the inverse of this. Fig. 4b–d displays units in which the response to the B tone is more dominant. Fig. 4b shows a complex pattern across FD. At an FD of 0 and 6 semitones neither tone produces strong responses. However, at an FD of 3 semitones the B tone response is stronger, whilst at large FDs (9, 12 semitones) the responses to the A tone are stronger. The A tone frequency is identical across all conditions, suggesting a suppression of the A tone response which depends on the frequency of the B tone. Fig. 4c and d shows neurons that are tuned to the B tone that is 6 and 12 semitones away respectively.

Across the entire population of neurons, at low PRs (2 Hz, 4 Hz) the changes in responses to B tones with FD generally reflected the responses to isolated tones. This broke down with the weaker responses displayed at high PRs (median Pearson correlations decrease with increasing PR: 0.86, 0.8, 0.66, 0.61, 0.55; p < .01, Friedman test). Thus, neurons which were not well tuned to the A tones generally gave results which are consistent with the hypothesis that tones that would be grouped into separate streams perceptually are represented in separate populations of neurons. Nevertheless, it is clear that responses to the different tones can interact in complex ways (Fig. 3C), which suggests that responses to tone sequences are a product of both the tuning of the neuron and a mutual suppression of the A and B tones.

### 3.4 A and B tones exhibit different rates of adaptation

An important feature of psychophysical auditory streaming is that the perception of two separate streams takes time to build up (around 5–10 s; Bregman, 1978; Anstis and Saida, 1985; Carlyon et al., 2001). One neural mechanism that has been posited to underlie this build-up is the multi-second adaptation of neural responses that occurs as a tone sequence proceeds (Micheyl et al., 2005; Snyder et al., 2006; Pressnitzer et al., 2008). Here we consider the time-course of adaptation in the population response to A and B tones throughout the sequence.

**Fig. 5:**
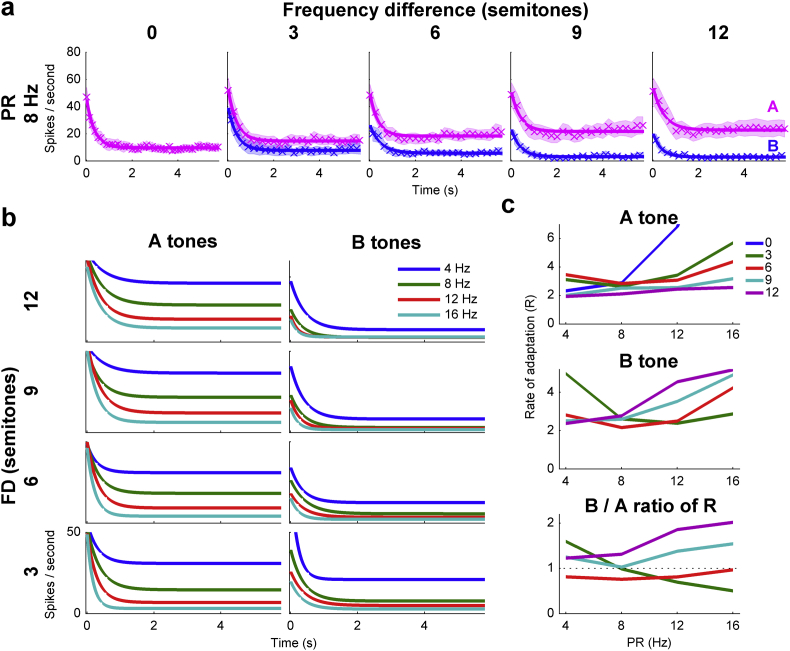
Variable rates and extents of adaptation were observed across the different PR and FD conditions. a) Mean spike count for each A and B tone fitted with decaying exponential functions for the 8 Hz conditions. SEMs are indicated by shaded patches around the crosses. b) Exponential fits for both the A and B tones for PRs from 4 to 16 Hz and semitone differences of 3–12 semitones. c) The rate of adaptation (R: see methods) for both the A and B tone fits increased as the PR was increased. However, the ratio of R shows that the B tone tended to adapt quicker than the A tone at faster PRs.

In the population of neurons investigated here, adaptation occurred in the responses to both the A and B tones (Fig. 5a; fitted with exponential functions). At all FDs, adaptation was complete within 2 s for both the A and B tones for PRs of 4 Hz and above; there was little or no adaptation for the 2 Hz condition (not shown). As the FD was increased, the B tone adapted more rapidly (Fig. 5c) and to a lower steady-state spike count (the 8 Hz condition is displayed in Fig. 5a). In contrast, and consistent with a release from the suppressive effect of the B tone on the A tone responses, an effect that accumulates during the sequence, adaptation of the A tone became slower and less profound as the FD was increased.

Changes in the mutual suppression of the A and B tone responses are also visible across PR (Fig. 5b). Both A and B tones adapted more rapidly as the PR was increased. However, the degree to which B tone responses adapted is largely constant at PRs above 8 Hz while A tone response adaptation was more gradual with increments in PR. The rate of adaptation increased as PR was increased (Fig. 5c), but it increased to a larger extent for the B tone responses as shown by the B/A ratio of adaptation rates (lower panel Fig. 5c). Thus, as the PR was increased, B tone adaptation became increasingly rapid compared to that of the A tone which led to the differential suppression of the A and B tones noted in the steady-state responses (Fig. 3).

### 3.5 Predictions of perceptual streaming from the neural population

Spike count measures indicate how neural activity changes with respect to stimulus properties such as PR and FD. However, to better relate neural responses to psychophysical streaming data we implemented a modification of a previous model based on signal detection theory (Micheyl et al., 2005; Pressnitzer et al., 2008). The aim of the model was to predict, from the weight of evidence in the neural representation, whether a one- or two-stream percept was more likely.

**Fig. 6:**
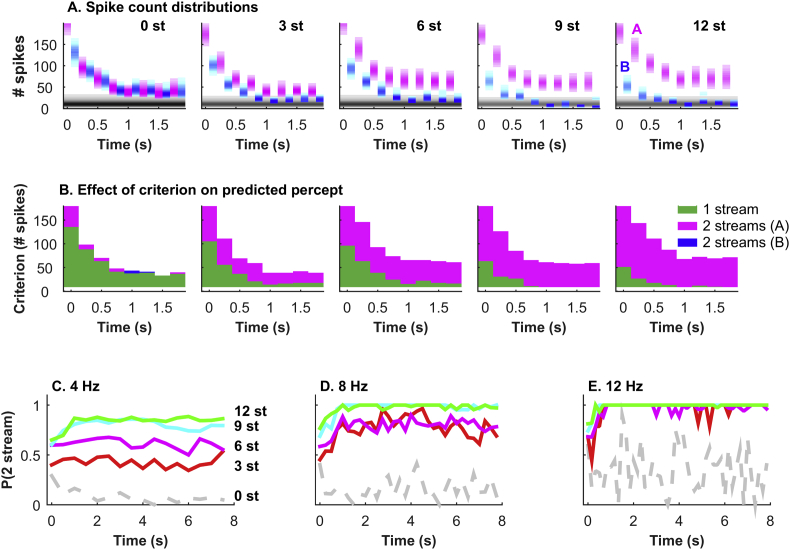
The SDT model. A. Population spike count distributions, tone by tone for A and B tones, for the first 0–2s of the tone sequences when PR is 8 Hz. The grey shaded horizontal bar shows the average spike count distribution in equivalent time windows when no tone is played. B. Distributions of decisions made as to whether tones belong to the same stream (green) or are segregated (2 streams; pink or blue), as a function of position in time during the sequence, and the applied decision criterion. The vertical axis indicates the criterion value or ‘threshold’ population spike count for considering a tone present. Coloured/shaded areas indicate when either or both tones produce more spikes than the criterion 75% or more of the time. White areas correspond to times and criterion when neither tone is detected, or false alarms in the absence of tones exceeds 25%, indicating tones are not distinguishable from background activity. C. Prediction of the probability of the tones being perceived as segregated, as a function of time (0–6 s), for all FDs and PRs of 4 Hz (left), 8 Hz (middle) and 12 Hz (right). This is calculated as the proportion of the criterion range (in Fig. 6B) over which the model predicts two streams will be perceived. Jagged lines at high PRs (12 Hz) indicate detection of either tone is unreliable so the chance of detecting 1 or both fluctuates from tone to tone. (For interpretation of the references to color in this figure legend, the reader is referred to the web version of this article.)

In Fig. 6A we display example population spike count distributions (see methods) in response to each A (pink) and B (blue) tone during the first 2 s of the ABAB sequences, presented at an 8 Hz PR. The vertical spread of the distributions indicates the variation in total spike count over the population across repeated presentations of the same sequence. The percept that is predicted from the neural population response depends on the differences in the distributions of spike counts in response to A and B tones. Additionally, significant overlap of the tone responses with the silent reference distribution (when that tone was omitted: gray shading near the horizontal axes; see methods) indicates that the tones were undetectable when compared with background neural activity (in this population of neurons tuned to the A tone frequency). If only A tones were represented in the neural responses, then the data suggest a two-stream percept (e.g. Fig. 6A, 12 semitones FD). If both tones were represented and produced similar responses then the model would predict a one-stream percept (e.g. Fig. 6A, 0 semitones FD). However, if both tones were represented, but the actual spike counts differed for the A and B tones, then the prediction is less clear (e.g. Fig. 6A, 6 semitones FD for the first second of the sequence).

To simulate a ‘decision’ as to whether the population response of neurons could be interpreted as evidence of a single stream or a two-stream percept, a threshold criterion was applied to the spike count distributions. This criterion was the only parameter in our model and was varied from low values, where the tones would be just distinguishable from background activity, to the highest criterion values where no response to any tone was represented (the criterion value can be visualized as a threshold horizontal line across the plots in Fig. 6A). Fig. 6B shows this for all FDs at 8 Hz PR. For each consecutive pair of tones (A first, B second), the plots indicate whether either or both tone responses exceed a given criterion value. Where criterion is exceeded by the responses to the A tone alone (pink) or the B tone alone (blue; unlikely), a two-stream percept is predicted. Where the criterion is exceeded by responses to both tones (green), a one-stream percept is predicted. It is clear that for different stimulus conditions and different positions within the sequence, the balance of the different predictions varies. Where, for most of the criterion range, only A tones produced responses above the criterion (e.g. Fig. 6B, PR 8 Hz, 12 semitones FD), then the neural representation was most consistent with a two-stream organization. Where the range of criterion values for which only one tone was detectable was very small (e.g. Fig. 6B, 0 semitones FD) whilst the range over which two tones were detectable was large, then the representation would suggest a one-stream percept.

Fig. 6C shows that the proportion of criterion values for which the A tone alone was detected (pink sections in Fig. 6B) increases with both PR and FD. The probability of a 2-stream neural representation also increases over the sequence duration, although in all cases the values are relatively unchanging after 2 s. Note that when tone responses are weak overall, such as when the FD is small and the PR is high, the proportion of one versus two-stream organizations is liable to fluctuate. In effect there is poor evidence that one could perceive either of the tones reliably from these responses (which seems unlikely).

**Fig. 7:**
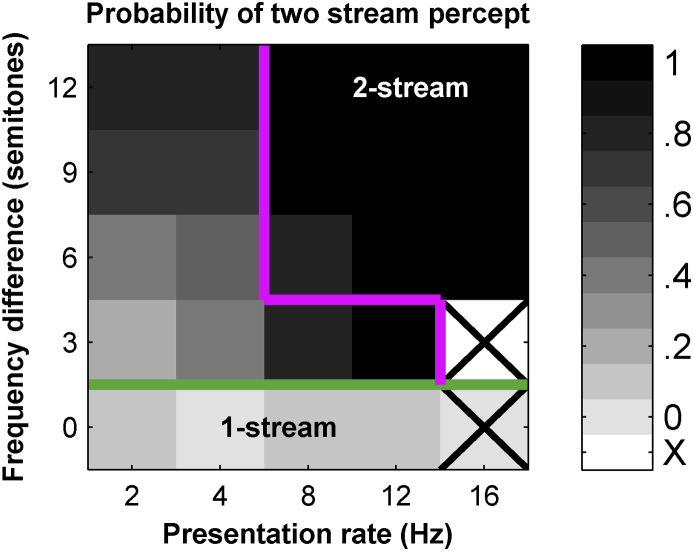
Overall predicted probability of perceiving two streams across all stimulus conditions, for the period 4–8s after the start of the sequence. Thick lines indicate the coherence (green/dark grey) and segregation (pink/light grey) boundaries seen in human psychophysics, with the region in between being the ambiguous region. The probability is calculated in the same manner as Fig. 6C–E. Thus it represents the proportion of criterion for which only 1 tone is reliably detected (compared with all criterion at which one or more tones are detectable). Crossed squares indicate conditions where the A tone was not reliably distinguished from the background at any criterion. (For interpretation of the references to color in this figure legend, the reader is referred to the web version of this article.)

To assess the overall predicted probability of perceiving two streams we considered the steady-state responses to all tones occurring between 4 and 8 s after commencement of the sequence (Fig. 7). This gave reliable predictions at all except the fastest PR for 0 and 3 semitones FD. When FD and PR were both large the neural representation predicted a 2-stream percept irrespective of the decision criterion. However, if the spike count distributions between the A and B tones were different then both one- and two-stream organizations are possible. This corresponds reasonably well to situations where either organization is possible perceptually, such as the ambiguous region bordered by the pink and green lines in Fig. 7.

## 4 Discussion

### 4.1 The influence of anesthesia

It is likely that anesthesia accentuates the effects of forward suppression in our data compared with responses in an awake animal. While urethane lowers average cortical firing rates (Albrecht and Davidowa, 1989; Capsius and Leppelsack, 1996; Syka et al., 2005), we are unaware of any studies directly comparing adaptation under urethane with awake responses. Ketamine significantly increases the duration of suppression by preceding stimuli in auditory cortex (Rennaker et al., 2007). However, unlike ketamine, which acts on excitatory NMDA receptors, or pentobarbitol which accentuates the effect of inhibitory GABA_A_ receptors, urethane anesthesia is produced by a modest effect on multiple receptor systems (Hara and Harris, 2002). The effects of Hypnorm, a combination of a mu-opioid agonist (fentanyl) and a dopamine antagonist (fluanisone), on cortical activity are less well documented.

Compared with responses to similar sequences of tones recorded from the auditory cortex of awake animals, our data showed weaker, less synchronized responses. Our un-adapted firing rates were lower (from Fig. 5; ∼40 spikes/sec; c.f. (at least 100 spikes/sec in Micheyl et al., 2005; Bee et al., 2010). Adaptation was also more complete. At 16 Hz, A tone responses were around 10% of the unadapted firing rate. At PRs greater than 2 Hz adaptation of A and B tones was complete in less than 2 s (Figs. 5b and 6C) across all FDs and the minimum rate of adaptation was over twice as fast as the maximum reported in awake starlings (Bee et al., 2010).

This strong adaptation also had an impact on the relative representation of A and B tones. In awake monkeys, differential suppression (as measured using the B/A ratio) consistently increased as the PR was increased up to 40 Hz (Fishman et al., 2001). The same trend was evident in our data up to 8 Hz, above which the B/A ratio increased. This was because the response to the A tone continued to reduce with increasing PR, whilst the response to the B tone had reached asymptote (see Fig. 3). This effect persisted when only considering units that phase locked at a given PR (10% of units at 16 Hz PR).

### 4.2 Forward suppression

Our data offers further evidence that frequency-tuned forward suppression plays a role in stream segregation (Fishman et al., 2001, 2004). Across the auditory pathway, forward suppression is often frequency-tuned and strongest when the preceding sound is in the center of the receptive field (Harris and Dallos, 1979; Brosch and Schreiner, 1997). In addition to an increasing suppression of B tone responses with FD and PR, we observed increasing responses to A tones as FD increased. This latter effect is consistent with a reduction of the suppressive effect of the B tone responses on the A tone responses as the B tone is moved away from the center of the neuron's receptive field, or a reduction in overall adaptation as the B tone stimulates the neuron less effectively. This effect contributed strongly to the representation of tones in our data at higher PRs, and is also evident in the cochlear nucleus (Pressnitzer et al., 2008). However, the competitive process described here is not a compete explanation of the effects of forward suppression. Forward suppression in some nuclei is ‘stimulus specific’ within a single neuron: the reduction in responses to tones in a sequence is strongest when the tone frequencies are matched, whether sequences are of two tones or many (e.g. Ulanovsky et al., 2003; Scholes et al., 2011). Further studies are required to understand the relationship between stimulus specific effects observed in other experiments and the competitive suppression observed here.

### 4.3 Signal detection model

Our signal detection analysis differed from previous studies in several respects. Previous work relied on responses to A tones being robust, and assumed that actual spike counts in response to the A tones did not influence perceptual organization (Micheyl et al., 2005; Pressnitzer et al., 2008; Bee et al., 2010). Our analysis considered the responses to A tones and B tones jointly, motivated by the observation that the A tone responses increased with increasing frequency difference.

Another key difference was that previous studies have either allowed the criterion to be a free parameter to fit data (Micheyl et al., 2005; Pressnitzer et al., 2008) or used a fixed criterion relative to spontaneous activity (Bee et al., 2010). It was clear from our data that a different criterion would have been required at each PR to account for differences across FD. We also felt that since perceptual organization is ambiguous and subject to non-sensory factors (van Noorden, 1977; Carlyon et al., 2001, 2003; Denham and Winkler, 2006; Pressnitzer and Hupe, 2006) it was worthwhile capturing the degree of ambiguity in the neural representation as well. Even if this neural ambiguity cannot be related to perceptual ambiguity, at least it offers an unbiased view of the evidence for organization in the neural representation.

In our data, responses adapted strongly at higher PRs. Thus we imposed a lower limit on the spike count criterion that ensured that tone responses were reliably distinguishable from silent gaps. This allowed us to take account of how reliably the A tone itself was represented and also indicated when A tones were represented, but B tone responses were indistinguishable from background activity.

### 4.4 Stream segregation across the auditory pathway

Our experimental model is the same as that used by Pressnitzer et al. (2008) to record from the cochlear nucleus, in terms of both species and anesthetic. Their stimuli were most similar to our own at a PR of 8 Hz. Both studies found an effect of FD on the output from a similar (but not identical) SDT model. However, in the cochlear nucleus the probability of predicting a two-stream percept rose gradually over the sequence duration, commensurate with the rate at which stream segregation builds perceptually. This was attributable to robust adaptation over a longer timescale than we observed in primary auditory cortex. Thus, our results do not show a simple inheritance of response properties from the brainstem.

The profusion of descending connections in the auditory pathway means that it is also possible that processing in auditory cortex, or other sub-cortical structures could modulate descending influences on the brainstem (Shore, 1998) or cochlea (Sridhar et al., 1995). Pressnitzer et al. (2008) speculated that this mechanism was behind the multi-second adaptation they observed in CN. Disentangling inheritance in this network is complicated. However, since we did not observe multi-second adaptation in our cortical responses, it seems unlikely that the multi-second adaptation observed in the cochlear nucleus under urethane/Hypnorm anesthesia can be due to gradual changes in descending modulation from the cortex. Instead, these datasets are consistent with multi-second adaptation being a property of either afferent processing occurring early in the auditory pathway, or a consequence of sub-cortical feedback. The effect of anesthesia on brainstem responses to tone sequences is likely to be fairly subtle (May and Sachs, 1992), and it seems likely that multi-second adaptation may well be robust in the brainstem of awake animals. This may be inherited by the awake auditory cortex, but it is hidden by strongly accentuated cortical adaptation under anesthesia. However, this remains speculative without recordings made during direct disruption of the efferent pathway. Additionally, it remains to be seen whether the effects of PR in the cochlear nucleus are consistent with perception.

### 4.5 Bottom up processes in stream segregation

Our study adds to the evidence that stimulus driven (primitive) processes consistent with perceptual stream segregation can be observed in neural responses. We also find that the neural evidence in favor of one or two streams can be ambiguous. This occurs in conditions where both A and B tones are represented clearly in the population responses, but the A and B tones elicit distinct firing rates. The degree of ambiguity varies with stimulus conditions in a way consistent with perception. One possibility is that this ambiguous cortical representation corresponds to the conditions over which perception is also ambiguous (van Noorden, 1977). Of course, this would manifest in different ways when attention is active. Evidence argues strongly against a static cortical representation (e.g. Fritz et al., 2003; Snyder and Alain, 2007; Paltoglou et al., 2011; Mesgarani and Chang, 2012). For example, a gain change applied in a frequency selective manner to cortical neurons might modify the ambiguity of this cortical representation in awake, attending conditions.

Our data are also not entirely consistent with the perceptual build-up of streaming since build-up was too rapid. Additionally, according to our analysis across all criterion values the evidence occasionally builds in favor of integration instead of segregation (for example at 2 Hz PR). This would contradict the perceptual data, where the perceptual organization at the beginning of a sequence is always integrated (Bregman, 1990). This could be interpreted as evidence in favor of higher level prediction-based processes. However, a more likely explanation relates to the strong and rapid adaptation, which we attribute in part to anesthesia.

Overall most of the trends evident in awake cortical recordings were evident in our data. The robustness of these qualitative effects in the face of clear quantitative changes argues in favor of them being hardwired, and a fairly immutable property of central auditory processing.
